# Partial Avulsion of Common Bile Duct and Duodenal Perforation in a Blunt Abdominal Trauma 

**Published:** 2010-12-01

**Authors:** Bilal Mirza, Lubna Ijaz, Shahid Iqbal, Afzal Sheikh

**Affiliations:** Department of Pediatric Surgery, The Children's Hospital and the Institute of Child Health Lahore, Pakistan

**Keywords:** Common bile duct, Avulsion, Blunt abdominal truama, Duodenal perforation

## Abstract

Complete or partial avulsion of common bile duct is a very rare injury following blunt abdominal trauma in children. A 7-year old boy presented to ER following blunt abdominal trauma by a moving motorcycle. X ray abdomen revealed free air under diaphragm and CT scan showed pancreatic contusion injury. At operation anterior wall of common bile duct (CBD) along with a 2mm rim of duodenal tissue on either side of anterior wall of CBD were found avulsed from the duodenum. The avulsed portion of CBD and duodenum were reanastomosed and a tube cholecystostomy performed. The patient had an uneventful recovery.

## INTRODUCTION

CBD avulsion in children, after a blunt abdominal trauma, is an uncommon injury. This is often associated with hepatic, duodenal, gastric and pancreatic injuries [1,2]. Avulsion of CBD may be complete or partial. In any case the presentation depends upon the extent of biliary peritonitis and associated injuries. Mostly the patients presented early within 2 to 4 days however delayed presentation has also been reported [3,4].

The preoperative diagnosis of CBD avulsion is never recognized preoperatively. A case of partial avulsion of CBD with duodenal perforation presenting with pneumoperitoneum is being reported which were successfully managed by primary repair. 

## CASE REPORT

A 7-year old boy was hit by a motorcycle in the abdomen. The patient was brought to our hospital, immediately after the incident, by emergency rescue service of the city. Patient was in obvious pain with pulse 130/min and respiratory rate 35/min. 

Patient was resuscitated. After stabilization an abdominal radiograph and ultrasound were requested. Abdominal radiograph revealed free air under diaphragm. Ultrasound of the abdomen showed a swollen pancreas with moderate free fluid in the peritoneal cavity. A CT scan of the abdomen was then performed that too delineated pancreatic injury (Fig. 1).

**Figure:**
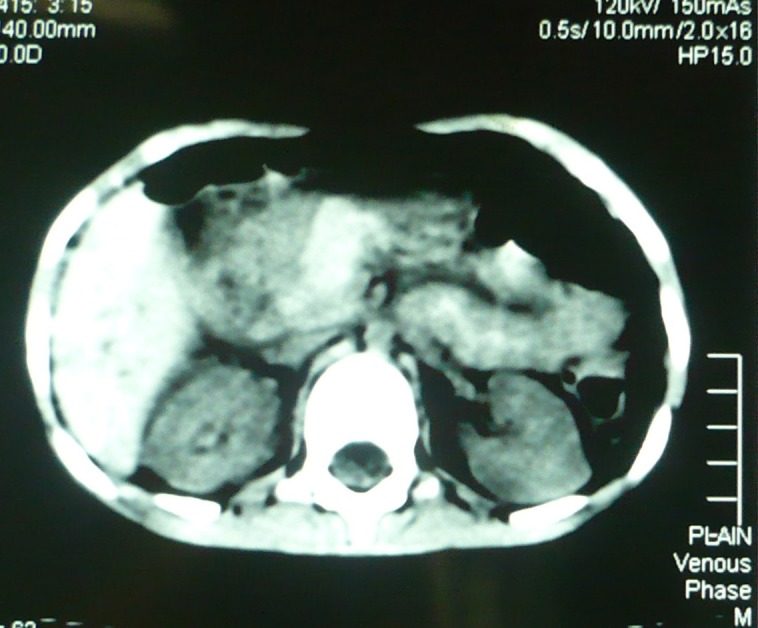
Figure 1: CT scan of abdomen showing pancreatic contusion injury; the pancreas is swollen and duodenal area is obscured by hypodense area.

At operation, about a liter of blood mixed with bile was drained from the peritoneal cavity. There was a bruise in the area of second part of duodenum with small amount of bile in the vicinity. Pancreas was edematous and swollen. Duodenum was mobilized and an avulsion of the anterior wall of CBD having a rim of duodenal tissue (2mm) on either side was noted that resulted in a rent of about 1cm in the duodenum. Bile was freely coming from the ampulla of Vater (Fig. 2,3). The partially avulsed CBD and duodenal rim were reanastomosed with duodenum in a single layer using interrupted extramucosal stitches in the long axis of the CBD. 

**Figure:**
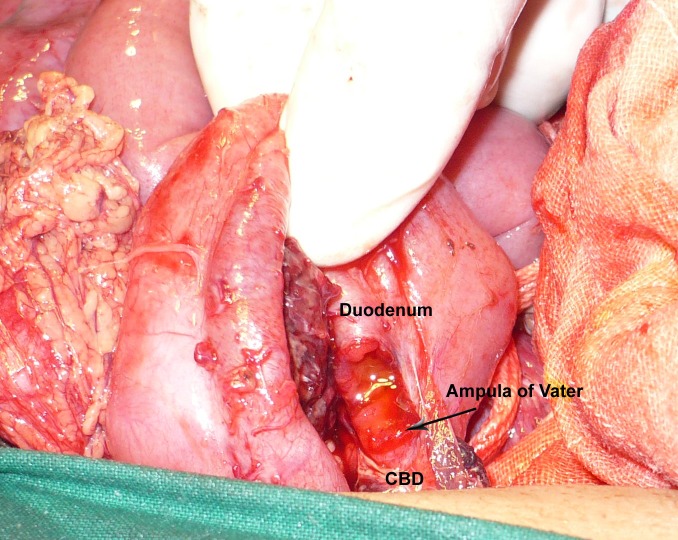
Figure 2: Operative view showing partial CBD avulsion.

**Figure:**
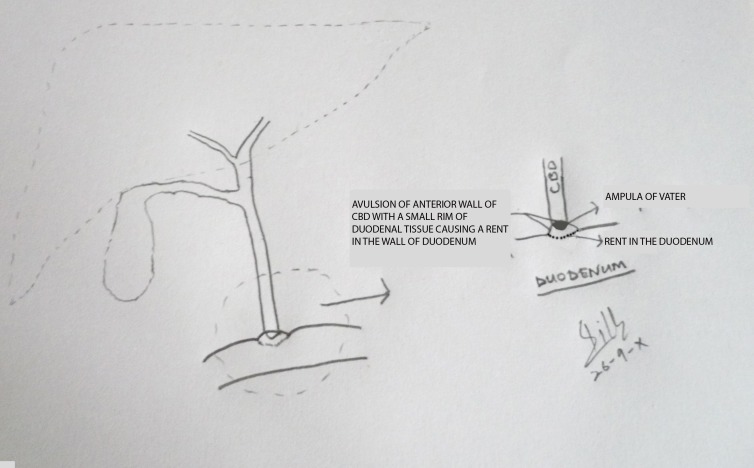
Figure 3: A line diagram illustrating the avulsion of anterior wall of CBD and duodenal rent.

A tube cholecystostomy was then performed to divert the bile flow. The postoperative recovery was uneventful. A tube cholangiogram was performed after two weeks that showed free passage of contrast into the duodenum (Fig. 4). Tube was removed and spontaneous closure of the cholecysto-cutaneous fistula occurred. Patient is doing well at a follow up of 6 months.

**Figure:**
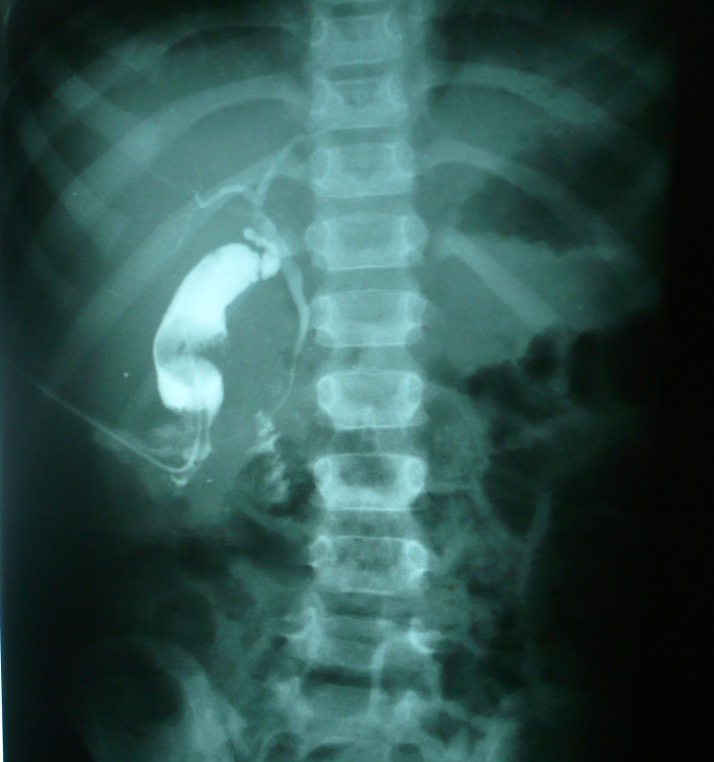
Figure 4: Tube cholangiogram showing free flow of contrast into the duodenum.

## DISCUSSION

The blunt abdominal injuries may result in significant insult to the various abdominal organs. Biliary tract injuries (gallbladder and CBD) are less frequently reported and occur in addition to other visceral injuries especially with liver, duodenum and pancreas. Isolated biliary tract injuries are extremely rare and limited to few case reports [1-4]. In our case the associated injuries were duodenal perforation and pancreatic contusion.

Patients with biliary tract injuries usually present early with signs of peritonitis and even shock, however, delayed presentation has also been reported with abdominal pain, jaundice and other vague symptoms [4-6].

The preoperative diagnosis of biliary tract injuries is not easy and in majority of reported cases it was made at operation. CBD may be avulsed, perforate or get contused following trauma. Rarely complete avulsion of ampulla of Vater is reported. Most of the CBD avulsion injuries are found at the level of pancreas, however, partial avulsion of CBD at the level of insertion into the duodenum has not been reported before. In our case the partial avulsion of CBD resulted in a tear in duodenum causing leakage of air and bile in the peritoneal cavity.

For CBD avulsion injuries the operative procedures include reinsertion into the duodenum (choledochoduodenostomy), choledocho-choledochal anastomosis, ligation of distal CBD and cholecysto-duodenal anastomosis, roux-en-y choledocho-jejunostomy and portoenterostomy and so on [1]. We re-stitched the duodenal rim and the anterior wall of CBD with duodenal wall in the long axis of the CBD, extramucosally, thus sparing the lumen of CBD. Tube cholecystostomy was also done for bile diversion. 

Pancreatic injuries may result in pancreatitis or as a late presentation pseudcyst may develop [7]. In our case no such complication occurred. The other concern is related to narrowing of repaired part of CBD. This is less likely in our case as posterior wall of CBD was intact, however a long term follow up is planned to observe this complication.

## Footnotes

**Source of Support:** Nil

**Conflict of Interest:** None declared
